# Supplementary material for: Evaluation and comparison of large language model responses to patient questions after diagnosis of high-risk human papillomavirus infection: an expert-rated digital patient education study
Source: Front Public Health. 2026 Jul 31;14:1915867. doi: 10.3389/fpubh.2026.1915867 (PMC13473150; doi:10.3389/fpubh.2026.1915867)
Supplement: Supplementary file 1 [file Supplementary_file_1.DOCX]

**Supplementary Figure S1. Mean total score by question and model.**

The figure shows question-level mean total scores for ChatGPT-5.5 Instant and DeepSeek-V3. The total score was calculated as the sum of the five Likert-domain scores and ranged from 5 to 25, with higher scores indicating better overall response quality.

**Supplementary Figure S2. Response stability across repeated generations by model.**

The figure shows the mean total scores across three repeated generations for each model. Error bars indicate standard errors. The total score was calculated as the sum of the five Likert-domain scores.

**Supplementary Table S1. Full AI-generated responses to patient-style questions**

**Q1. I tested positive for high-risk HPV. Does this mean I will definitely develop cervical cancer in the future?**

| **Response** | **Full AI-generated response (English translation)** |
| --- | --- |
| CA1 ChatGPT-5.5 Instant Repeat 1 | Most high-risk HPV infections do not develop into cervical cancer, and many people may become negative within 1-2 years. The real danger is persistent infection for many years without regular follow-up. |
| CA2 ChatGPT-5.5 Instant Repeat 2 | High-risk HPV positivity does not mean that you will definitely develop cervical cancer. Most women clear the virus spontaneously within 1-2 years. Only a small proportion of women with persistent infection for many years and without timely follow-up may develop precancerous lesions or cancer. |
| CA3 ChatGPT-5.5 Instant Repeat 3 | High-risk HPV positivity does not mean that you will definitely develop cervical cancer. Most infections are cleared by the body, and only a small proportion that persists for many years may develop into precancerous lesions or cancer. Regular follow-up as advised by your doctor is most important. |
| DA1 DeepSeek-V3 Repeat 1 | No. Most HPV infections are like a 'cervical cold' and disappear on their own within 1-2 years. Only long-term persistent infection may develop into precancerous lesions, and this process usually takes 5-10 years, leaving sufficient time for intervention. |
| DA2 DeepSeek-V3 Repeat 2 | No. The vast majority of infections are cleared by the body's own immune system within 1-2 years. Only long-term persistent infection may slowly progress, and this process usually takes 5-10 years. |
| DA3 DeepSeek-V3 Repeat 3 | No. Just as being infected with the influenza virus does not mean getting pneumonia, progression from infection to cancer usually takes 5-10 years, so you have enough time to block it. |

**Q2. Is HPV16 or HPV18 positivity serious? Do I definitely need colposcopy?**

| **Response** | **Full AI-generated response (English translation)** |
| --- | --- |
| CA1 ChatGPT-5.5 Instant Repeat 1 | HPV16 and HPV18 are high-risk types for cervical cancer and indeed need more attention than other types. Many guidelines recommend further colposcopy, but whether it must be done depends on the TCT result, age, and the doctor's judgment. |
| CA2 ChatGPT-5.5 Instant Repeat 2 | HPV16 and HPV18 are higher-risk types, but they do not necessarily lead to cancer. Whether colposcopy should be done depends on the TCT result, age, and the doctor's assessment; in many cases, doctors will recommend further examination. |
| CA3 ChatGPT-5.5 Instant Repeat 3 | HPV16 and HPV18 are higher-risk types, but they do not necessarily lead to cancer. Many doctors will recommend further colposcopy, especially when combined with an abnormal TCT result, to examine the cervix more carefully. |
| DA1 DeepSeek-V3 Repeat 1 | These two types carry the highest risk and account for about 70% of cervical cancers. If positive, doctors usually recommend colposcopy for further examination. Do not delay. |
| DA2 DeepSeek-V3 Repeat 2 | These are the two highest-risk types, and doctors will be more vigilant. Direct colposcopy for further examination is usually recommended. |
| DA3 DeepSeek-V3 Repeat 3 | This needs attention, but it does not mean that you are seriously ill. They cause more than 70% of cervical cancers, so doctors recommend magnified observation with colposcopy, which is like a more careful screening to avoid missed diagnosis. |

**Q3. I am positive for high-risk HPV, but my TCT result is normal. Do I need treatment? When should I be retested?**

| **Response** | **Full AI-generated response (English translation)** |
| --- | --- |
| CA1 ChatGPT-5.5 Instant Repeat 1 | When high-risk HPV is positive but TCT is normal, immediate treatment is often not needed; the focus is regular follow-up. A common approach is repeat HPV/TCT testing after 1 year, while some patients positive for HPV16 or HPV18 may be advised to undergo colposcopy directly. |
| CA2 ChatGPT-5.5 Instant Repeat 2 | If HPV is positive but TCT is normal, many people do not need treatment for the time being; the focus is regular follow-up. Repeat HPV and TCT testing is generally recommended in about 1 year, with the exact timing arranged by the doctor. |
| CA3 ChatGPT-5.5 Instant Repeat 3 | If HPV is positive but TCT is normal, many people do not need treatment for the time being and are mainly followed by observation. Repeat HPV and TCT testing is generally recommended in about 1 year, with the exact timing arranged by the doctor. |
| DA1 DeepSeek-V3 Repeat 1 | There is no specific medicine. A normal TCT means that there is no cellular lesion, and clearance mainly depends on immunity. Repeat HPV plus TCT testing in 6-12 months is generally sufficient. |
| DA2 DeepSeek-V3 Repeat 2 | There is no specific medicine. The main approach is to improve immunity, maintain regular routines, and exercise. Retesting is generally done after 12 months. |
| DA3 DeepSeek-V3 Repeat 3 | No medication is needed; rely on immunity. There is no specific medicine. A simple infection is cleared by the body itself, and combined retesting (TCT plus HPV) is recommended after 12 months. Normal daily life can continue during this period. |

**Q4. What should I do if HPV remains positive and does not clear? Is there any medicine that can completely eliminate HPV?**

| **Response** | **Full AI-generated response (English translation)** |
| --- | --- |
| CA1 ChatGPT-5.5 Instant Repeat 1 | There is currently no specific medicine that can 'completely clear HPV.' Most treatments treat lesions rather than directly killing the virus. If HPV remains positive for a long time, regular follow-up and monitoring whether the cervix has changed are more important. |
| CA2 ChatGPT-5.5 Instant Repeat 2 | Persistent HPV positivity does not necessarily mean that the condition is serious. At present, there is no specific medicine that can 'completely kill' HPV. The key is regular follow-up, timely treatment if lesions are found, and maintaining good lifestyle habits. |
| CA3 ChatGPT-5.5 Instant Repeat 3 | Persistent HPV positivity does not necessarily mean that the condition is serious. At present, there is no specific medicine that can 'completely eradicate' HPV. The focus is regular follow-up and timely treatment if abnormalities are found. Keeping regular sleep patterns and reducing staying up late may also help. |
| DA1 DeepSeek-V3 Repeat 1 | There is no oral medicine that can 'eradicate' the virus. The focus is regular monitoring and timely treatment of lesions if found, such as cervical conization. Do not waste money on products claiming to make HPV 'turn negative.' |
| DA2 DeepSeek-V3 Repeat 2 | Be patient; most infections will become negative. If HPV remains positive for several years, the doctor will treat lesions according to the situation, rather than treating the virus itself. |
| DA3 DeepSeek-V3 Repeat 3 | At present, no oral or topical medicine can directly kill the HPV virus. Doctors will monitor persistent infection and treat it if lesions appear; as long as there are no lesions, continue to rely on immunity and wait for clearance. |

**Q5. My doctor said I have ASC-US or LSIL. How is this related to HPV infection? Is it serious?**

| **Response** | **Full AI-generated response (English translation)** |
| --- | --- |
| CA1 ChatGPT-5.5 Instant Repeat 1 | ASC-US means 'mild abnormality but atypical,' and LSIL means 'low-grade lesion.' They are often related to HPV infection. Many LSIL cases also recover on their own and do not mean cancer, but follow-up is needed. |
| CA2 ChatGPT-5.5 Instant Repeat 2 | ASC-US and LSIL are mild abnormalities of cervical cells and are often related to HPV infection. Most cases are not serious, and some can recover spontaneously, but follow-up on schedule is needed and colposcopy should be performed if necessary. |
| CA3 ChatGPT-5.5 Instant Repeat 3 | ASC-US and LSIL indicate mild abnormalities in cervical cells and are often related to HPV infection. Most cases are not serious, and some can recover spontaneously, but follow-up on schedule is needed and colposcopy should be performed if necessary. |
| DA1 DeepSeek-V3 Repeat 1 | These are slight or low-grade cellular abnormalities, mostly caused by HPV, and are not serious. About 60% will return to normal spontaneously, and the doctor may recommend observation or colposcopy. |
| DA2 DeepSeek-V3 Repeat 2 | These are mild abnormalities on TCT and are mostly related to HPV infection. They are usually not serious, and many recover on their own. |
| DA3 DeepSeek-V3 Repeat 3 | They are related, and they are not serious. This means that the virus has caused slight cellular changes, but it is far from cancer. ASC-US is like the cells 'caught a cold,' and LSIL is 'mild inflammation'; many cases can heal on their own, so follow the doctor's advice for follow-up. |

**Q6. Can HPV infection clear on its own? How long does it usually take?**

| **Response** | **Full AI-generated response (English translation)** |
| --- | --- |
| CA1 ChatGPT-5.5 Instant Repeat 1 | HPV infection is very common, especially after sexual activity begins. Most infections clear on their own, usually in about 1-2 years, but a small proportion persist. |
| CA2 ChatGPT-5.5 Instant Repeat 2 | HPV infection may clear on its own. Most young women become negative within 1-2 years, while a small proportion remain positive for longer, so regular follow-up is important. |
| CA3 ChatGPT-5.5 Instant Repeat 3 | Many HPV infections clear on their own, commonly becoming negative within 1-2 years. Younger people and those with better immune status are more likely to clear the virus. |
| DA1 DeepSeek-V3 Repeat 1 | Yes. About 80%-90% of HPV infections are cleared by the immune system within 1-2 years. The clearance rate is higher in young women. |
| DA2 DeepSeek-V3 Repeat 2 | Yes. About 80%-90% of people clear the virus through immunity within 1-2 years. |
| DA3 DeepSeek-V3 Repeat 3 | Yes, it is very common. About 80%-90% of people clear the virus through immunity within 1-2 years, just as the body clears a cold virus. |

**Q7. Can I have sex after HPV infection? Will it be transmitted to my partner? Do I need to use condoms?**

| **Response** | **Full AI-generated response (English translation)** |
| --- | --- |
| CA1 ChatGPT-5.5 Instant Repeat 1 | After HPV positivity, sex is usually still allowed. HPV can be transmitted to a partner, and condom use can reduce the risk of transmission and reinfection, but it cannot prevent it 100%. |
| CA2 ChatGPT-5.5 Instant Repeat 2 | Sex is allowed after HPV positivity, but there is a possibility of transmission to the partner. Having a stable partner and using condoms are recommended to reduce the risk of transmission, although condoms cannot prevent it 100%. |
| CA3 ChatGPT-5.5 Instant Repeat 3 | Sex is generally still allowed after HPV positivity, but it may be transmitted to the partner. Having a stable partner and using condoms are recommended to reduce the risk of transmission and reinfection. |
| DA1 DeepSeek-V3 Repeat 1 | Yes, but condom use is recommended. It can reduce the risk of transmission, but it cannot block HPV completely. Transmission to a partner generally does not cause serious disease, so there is no need for excessive anxiety. |
| DA2 DeepSeek-V3 Repeat 2 | Yes, but condom use is recommended to reduce the viral load. |
| DA3 DeepSeek-V3 Repeat 3 | You can have sex, but condoms must be used throughout. They can greatly reduce the viral load, help you become negative, and protect your partner from repeated infection. |

**Q8. Does my partner need HPV testing or treatment?**

| **Response** | **Full AI-generated response (English translation)** |
| --- | --- |
| CA1 ChatGPT-5.5 Instant Repeat 1 | Male partners usually do not require routine screening. If there are genital warts, abnormal symptoms, or high-risk behaviors, they can consult a urology or dermatology department. |
| CA2 ChatGPT-5.5 Instant Repeat 2 | Male partners generally do not have routine HPV screening. If there are no obvious warts or symptoms, special treatment is usually not needed, but hygiene should be maintained and multiple sexual partners avoided. |
| CA3 ChatGPT-5.5 Instant Repeat 3 | Most male partners have no obvious symptoms and generally do not need routine treatment. If warts or abnormal symptoms appear, they should go to the hospital for examination. Both partners should pay attention to hygiene and protection. |
| DA1 DeepSeek-V3 Repeat 1 | No. There is no good testing method for men, and they will not develop cancer because of it. There is no need to ask your partner to undergo special testing or treatment. |
| DA2 DeepSeek-V3 Repeat 2 | No. There is no good way to treat men, and most men will clear it on their own. |
| DA3 DeepSeek-V3 Repeat 3 | Men usually do not need it. Testing in men has a high false-negative rate, and even if infected, cancer is extremely rare. He only needs to seek medical care if he has genital warts. |

**Q9. If I have already been infected with HPV, is HPV vaccination still meaningful?**

| **Response** | **Full AI-generated response (English translation)** |
| --- | --- |
| CA1 ChatGPT-5.5 Instant Repeat 1 | After HPV infection, HPV vaccination is still meaningful because the vaccine can still prevent other types that you have not yet been infected with. |
| CA2 ChatGPT-5.5 Instant Repeat 2 | Even if you have already been infected with HPV, vaccination is still meaningful. This is because the vaccine can prevent other types that have not yet been acquired and may also reduce the risk of future reinfection. |
| CA3 ChatGPT-5.5 Instant Repeat 3 | After HPV infection, HPV vaccination is still meaningful. The vaccine cannot clear the type that has already infected you, but it can prevent infection with other types and help reduce future risk. |
| DA1 DeepSeek-V3 Repeat 1 | It is meaningful. It can prevent other high-risk HPV types that you have not been infected with, reducing future reinfection and recurrence risks. |
| DA2 DeepSeek-V3 Repeat 2 | It is meaningful! It can prevent other types that you have not been infected with. |
| DA3 DeepSeek-V3 Repeat 3 | It is very meaningful! You are unlikely to have been infected with all subtypes, and the vaccine can still protect you against other high-risk or low-risk subtypes that you have not acquired. |

**Q10. Will HPV infection affect pregnancy planning, pregnancy, or the fetus?**

| **Response** | **Full AI-generated response (English translation)** |
| --- | --- |
| CA1 ChatGPT-5.5 Instant Repeat 1 | Simple HPV infection generally does not affect pregnancy or pregnancy planning and usually does not affect the fetus. The key is to confirm before pregnancy whether there are obvious cervical lesions. |
| CA2 ChatGPT-5.5 Instant Repeat 2 | Simple HPV infection usually does not significantly affect pregnancy or the fetus. Most women can plan pregnancy and become pregnant normally, but cervical examinations should be done on schedule. |
| CA3 ChatGPT-5.5 Instant Repeat 3 | HPV usually does not affect pregnancy, pregnancy planning, or fetal development. Most women can become pregnant and give birth normally, so there is no need for excessive worry. |
| DA1 DeepSeek-V3 Repeat 1 | Simple infection does not affect pregnancy planning or the fetus. However, if cervical surgery has been performed for high-grade lesions, such as conization, the risk of preterm birth may increase; after surgery, please inform the obstetrician when planning pregnancy. |
| DA2 DeepSeek-V3 Repeat 2 | It generally does not affect pregnancy. However, if the TCT result is severe and treatment is needed, treatment may be recommended before pregnancy. |
| DA3 DeepSeek-V3 Repeat 3 | HPV does not affect egg quality, sperm passage, or fetal development. As long as there are no cervical lesions requiring surgery, pregnancy can be planned normally. |

**Q11. What should I do if HPV positivity is found during pregnancy? Can it be treated?**

| **Response** | **Full AI-generated response (English translation)** |
| --- | --- |
| CA1 ChatGPT-5.5 Instant Repeat 1 | If HPV positivity is found during pregnancy, most cases are managed by observation and follow-up. Active treatment is usually not performed during pregnancy unless a high-grade lesion is suspected. |
| CA2 ChatGPT-5.5 Instant Repeat 2 | If HPV positivity is found during pregnancy, most cases are managed by observation and follow-up, and treatment is generally not urgent. The doctor will decide whether further examination is needed based on the condition of the cervix. |
| CA3 ChatGPT-5.5 Instant Repeat 3 | If HPV positivity is found during pregnancy, most cases are managed by observation and repeat testing, and treatment is generally not urgent. The doctor will decide whether further examination is needed based on the condition of the cervix. |
| DA1 DeepSeek-V3 Repeat 1 | In the vast majority of cases, repeat testing is done after delivery. Unless cancer is suspected, treatment is generally not performed. After delivery, immunity recovers, and many people become negative. |
| DA2 DeepSeek-V3 Repeat 2 | Do not panic; it generally will not be transmitted to the baby. Repeat testing can be done after delivery. |
| DA3 DeepSeek-V3 Repeat 3 | Usually, only observation is needed and no treatment is given. Hormonal changes during pregnancy may temporarily worsen it, but it often regresses after delivery. The doctor will reassess after delivery; unless cancer is suspected, no treatment is performed. |

**Q12. What should I pay attention to in daily life after testing positive for HPV? Do staying up late, stress, and immunity matter?**

| **Response** | **Full AI-generated response (English translation)** |
| --- | --- |
| CA1 ChatGPT-5.5 Instant Repeat 1 | Maintaining regular routines, reducing staying up late, smoking, and long-term stress may help immune status. Although this cannot guarantee HPV clearance, it is beneficial for recovery. |
| CA2 ChatGPT-5.5 Instant Repeat 2 | After HPV positivity, you should try to stay up late less, reduce stress, quit smoking, and maintain regular routines. Long-term fatigue and reduced immunity may be unfavorable for viral clearance. |
| CA3 ChatGPT-5.5 Instant Repeat 3 | After HPV positivity, it is recommended to stay up late less, avoid smoking, eat a balanced diet, and exercise regularly. Long-term stress and poor sleep may affect the body's ability to clear the virus. |
| DA1 DeepSeek-V3 Repeat 1 | Do not stay up late, quit smoking including secondhand smoke, reduce stress, exercise appropriately, and maintain a balanced diet. Immunity is the best 'medicine' for clearing HPV. |
| DA2 DeepSeek-V3 Repeat 2 | Do not stay up late, do not be anxious, maintain balanced nutrition, and exercise more. Immunity is the best medicine. |
| DA3 DeepSeek-V3 Repeat 3 | They have a major impact and are central to clearing the virus. Do the following: sleep before 11 p.m., keep a relaxed mindset, do sweat-inducing exercise three times a week, stop smoking and limit alcohol, and eat a balanced high-protein diet. Good immunity is your 'specific medicine.' |

**Q13. What do CIN1, CIN2, and CIN3 mean? Do they all require surgery?**

| **Response** | **Full AI-generated response (English translation)** |
| --- | --- |
| CA1 ChatGPT-5.5 Instant Repeat 1 | CIN1, CIN2, and CIN3 indicate progressively increasing degrees of cervical lesions. Many CIN1 cases can be observed; CIN2 and CIN3 require more active evaluation and sometimes treatments such as conization. |
| CA2 ChatGPT-5.5 Instant Repeat 2 | CIN1, CIN2, and CIN3 represent progressively increasing degrees of cervical lesions. Many CIN1 cases recover spontaneously; CIN2 and CIN3 have a higher risk of cancer progression, and whether surgery is needed depends on age, fertility needs, and the doctor's judgment. |
| CA3 ChatGPT-5.5 Instant Repeat 3 | CIN1, CIN2, and CIN3 indicate progressively increasing degrees of cervical lesions. Many CIN1 cases recover spontaneously; CIN2 and CIN3 have a higher risk of cancer progression and often require further treatment or surgery. |
| DA1 DeepSeek-V3 Repeat 1 | These are grades of cervical precancerous lesions. CIN1 is usually observed, and many cases regress; for CIN2/CIN3, doctors usually recommend a minor procedure, such as LEEP or conization, to remove the lesion, with a high cure rate. |
| DA2 DeepSeek-V3 Repeat 2 | These are grades of precancerous lesions. CIN1 is mostly observed, while CIN2/3 usually requires minor surgical removal. They are not cancer. |
| DA3 DeepSeek-V3 Repeat 3 | They are grades 1, 2, and 3 of cervical precancerous lesions. CIN1: most cases heal on their own, like a temporary drop in grades that can catch up, so observation is sufficient. CIN2/3: high-grade lesions, and doctors will recommend a minor procedure such as conization to remove the lesion area, with an extremely high cure rate. |

**Q14. What should I do if I have bleeding after sex, abnormal vaginal bleeding, or abnormal discharge?**

| **Response** | **Full AI-generated response (English translation)** |
| --- | --- |
| CA1 ChatGPT-5.5 Instant Repeat 1 | If bleeding after sex, abnormal vaginal bleeding, or clearly abnormal discharge occurs, seek medical care as soon as possible instead of only waiting for follow-up. |
| CA2 ChatGPT-5.5 Instant Repeat 2 | If bleeding after sex, abnormal vaginal bleeding, increased discharge, or odor occurs, go to a gynecologist as soon as possible and do not rely only on self-observation. |
| CA3 ChatGPT-5.5 Instant Repeat 3 | If bleeding after sex, abnormal vaginal bleeding, markedly increased discharge, or odor occurs, go for gynecologic follow-up as soon as possible and do not delay. |
| DA1 DeepSeek-V3 Repeat 1 | Please seek medical care as soon as possible. This is a warning sign that needs attention. The doctor will perform a gynecologic examination and colposcopy to determine the cause of bleeding and manage it. |
| DA2 DeepSeek-V3 Repeat 2 | See a doctor as soon as possible and let the doctor examine the cervix. |
| DA3 DeepSeek-V3 Repeat 3 | Go to the gynecology department immediately. This is not a minor issue, but there is no need to panic. It may be cervical inflammation, a polyp, or a lesion that needs treatment. Let the doctor perform a gynecologic examination and cervical screening to clarify the cause. |

**Q15. I am very anxious and worried that HPV will turn into cancer. What should I do first?**

| **Response** | **Full AI-generated response (English translation)** |
| --- | --- |
| CA1 ChatGPT-5.5 Instant Repeat 1 | The most important thing now is not excessive panic, but timely follow-up, colposcopy when necessary, and not being lost to follow-up. Cervical cancer usually develops slowly, and regular follow-up can greatly reduce the risk. |
| CA2 ChatGPT-5.5 Instant Repeat 2 | The most important thing now is not excessive fear, but timely follow-up. HPV-related cervical cancer usually develops slowly. With regular follow-up, most problems can be found and managed early. |
| CA3 ChatGPT-5.5 Instant Repeat 3 | The most important thing now is: do not panic excessively, and attend follow-up on time. HPV is very common, and most cases do not develop into cancer. With regular follow-up and timely management of abnormalities, the risk can usually be controlled. |
| DA1 DeepSeek-V3 Repeat 1 | 1) Stay calm and tell yourself, 'This is not cancer.' 2) Take the report to a gynecologist at a regular hospital and make a plan for follow-up or colposcopy. 3) Return to normal life and do not think about it every day. The vast majority of people will ultimately not develop cancer. |
| DA2 DeepSeek-V3 Repeat 2 | Trust your doctor and undergo follow-up or colposcopy as required. The vast majority of people are fine, and anxiety instead reduces immunity. |
| DA3 DeepSeek-V3 Repeat 3 | Stop being anxious and live seriously. You only have one more annual check-up task than others. By following medical advice for follow-up and improving immunity, you firmly hold the initiative. |

Note: The original prompts and AI-generated responses were in Chinese. The table presents faithful English translations without changing the original meaning. TCT refers to the ThinPrep cytologic test.

**Supplementary Table S2. Raw expert-rating data for all AI-generated responses**

| **No.** | **Expert** | **Question** | **Model** | **Repeat** | **Response ID** | **Accuracy** | **Safety** | **Guideline concordance** | **Completeness** | **Understandability** | **Major error** | **Potential harm** |
| --- | --- | --- | --- | --- | --- | --- | --- | --- | --- | --- | --- | --- |
| 1 | E1 | Q1 | ChatGPT-5.5 Instant | 1 | CA1 | 4 | 3 | 3 | 3 | 3 | No | No |
| 2 | E2 | Q1 | ChatGPT-5.5 Instant | 1 | CA1 | 4 | 4 | 3 | 4 | 4 | No | No |
| 3 | E3 | Q1 | ChatGPT-5.5 Instant | 1 | CA1 | 5 | 4 | 3 | 4 | 4 | No | No |
| 4 | E4 | Q1 | ChatGPT-5.5 Instant | 1 | CA1 | 5 | 4 | 4 | 4 | 3 | No | No |
| 5 | E5 | Q1 | ChatGPT-5.5 Instant | 1 | CA1 | 4 | 5 | 4 | 4 | 5 | No | No |
| 6 | E1 | Q1 | ChatGPT-5.5 Instant | 2 | CA2 | 5 | 5 | 5 | 5 | 5 | No | No |
| 7 | E2 | Q1 | ChatGPT-5.5 Instant | 2 | CA2 | 5 | 5 | 4 | 5 | 5 | No | No |
| 8 | E3 | Q1 | ChatGPT-5.5 Instant | 2 | CA2 | 5 | 5 | 4 | 4 | 5 | No | No |
| 9 | E4 | Q1 | ChatGPT-5.5 Instant | 2 | CA2 | 4 | 4 | 4 | 4 | 5 | No | No |
| 10 | E5 | Q1 | ChatGPT-5.5 Instant | 2 | CA2 | 5 | 3 | 5 | 5 | 5 | No | No |
| 11 | E1 | Q1 | ChatGPT-5.5 Instant | 3 | CA3 | 4 | 4 | 4 | 4 | 4 | No | No |
| 12 | E2 | Q1 | ChatGPT-5.5 Instant | 3 | CA3 | 5 | 5 | 5 | 4 | 5 | No | No |
| 13 | E3 | Q1 | ChatGPT-5.5 Instant | 3 | CA3 | 5 | 4 | 3 | 4 | 5 | No | No |
| 14 | E4 | Q1 | ChatGPT-5.5 Instant | 3 | CA3 | 4 | 4 | 4 | 4 | 4 | No | No |
| 15 | E5 | Q1 | ChatGPT-5.5 Instant | 3 | CA3 | 4 | 4 | 4 | 4 | 4 | No | No |
| 16 | E1 | Q1 | DeepSeek-V3 | 1 | DA1 | 4 | 3 | 3 | 3 | 4 | No | No |
| 17 | E2 | Q1 | DeepSeek-V3 | 1 | DA1 | 4 | 4 | 3 | 5 | 5 | No | No |
| 18 | E3 | Q1 | DeepSeek-V3 | 1 | DA1 | 4 | 4 | 3 | 5 | 5 | No | No |
| 19 | E4 | Q1 | DeepSeek-V3 | 1 | DA1 | 4 | 4 | 4 | 5 | 5 | No | No |
| 20 | E5 | Q1 | DeepSeek-V3 | 1 | DA1 | 5 | 3 | 5 | 5 | 5 | No | No |
| 21 | E1 | Q1 | DeepSeek-V3 | 2 | DA2 | 5 | 3 | 4 | 4 | 4 | No | No |
| 22 | E2 | Q1 | DeepSeek-V3 | 2 | DA2 | 3 | 5 | 3 | 4 | 5 | No | No |
| 23 | E3 | Q1 | DeepSeek-V3 | 2 | DA2 | 3 | 4 | 3 | 4 | 5 | No | No |
| 24 | E4 | Q1 | DeepSeek-V3 | 2 | DA2 | 4 | 4 | 4 | 4 | 5 | No | No |
| 25 | E5 | Q1 | DeepSeek-V3 | 2 | DA2 | 4 | 3 | 4 | 4 | 4 | No | No |
| 26 | E1 | Q1 | DeepSeek-V3 | 3 | DA3 | 3 | 3 | 3 | 3 | 5 | No | No |
| 27 | E2 | Q1 | DeepSeek-V3 | 3 | DA3 | 4 | 4 | 3 | 4 | 5 | No | No |
| 28 | E3 | Q1 | DeepSeek-V3 | 3 | DA3 | 4 | 4 | 3 | 4 | 5 | No | No |
| 29 | E4 | Q1 | DeepSeek-V3 | 3 | DA3 | 4 | 4 | 3 | 4 | 5 | No | No |
| 30 | E5 | Q1 | DeepSeek-V3 | 3 | DA3 | 3 | 3 | 3 | 3 | 4 | No | No |
| 31 | E1 | Q10 | ChatGPT-5.5 Instant | 1 | CA1 | 5 | 5 | 5 | 4 | 5 | No | No |
| 32 | E2 | Q10 | ChatGPT-5.5 Instant | 1 | CA1 | 5 | 5 | 3 | 5 | 5 | No | No |
| 33 | E3 | Q10 | ChatGPT-5.5 Instant | 1 | CA1 | 5 | 4 | 3 | 4 | 5 | No | No |
| 34 | E4 | Q10 | ChatGPT-5.5 Instant | 1 | CA1 | 4 | 4 | 4 | 4 | 4 | No | No |
| 35 | E5 | Q10 | ChatGPT-5.5 Instant | 1 | CA1 | 4 | 5 | 5 | 4 | 4 | No | No |
| 36 | E1 | Q10 | ChatGPT-5.5 Instant | 2 | CA2 | 4 | 4 | 4 | 4 | 4 | No | No |
| 37 | E2 | Q10 | ChatGPT-5.5 Instant | 2 | CA2 | 4 | 4 | 4 | 4 | 5 | No | No |
| 38 | E3 | Q10 | ChatGPT-5.5 Instant | 2 | CA2 | 4 | 4 | 3 | 4 | 5 | No | No |
| 39 | E4 | Q10 | ChatGPT-5.5 Instant | 2 | CA2 | 4 | 5 | 4 | 5 | 5 | No | No |
| 40 | E5 | Q10 | ChatGPT-5.5 Instant | 2 | CA2 | 5 | 3 | 5 | 4 | 3 | No | No |
| 41 | E1 | Q10 | ChatGPT-5.5 Instant | 3 | CA3 | 4 | 4 | 4 | 4 | 4 | No | No |
| 42 | E2 | Q10 | ChatGPT-5.5 Instant | 3 | CA3 | 4 | 4 | 4 | 4 | 4 | No | No |
| 43 | E3 | Q10 | ChatGPT-5.5 Instant | 3 | CA3 | 3 | 3 | 3 | 3 | 4 | No | No |
| 44 | E4 | Q10 | ChatGPT-5.5 Instant | 3 | CA3 | 4 | 4 | 4 | 4 | 4 | No | No |
| 45 | E5 | Q10 | ChatGPT-5.5 Instant | 3 | CA3 | 4 | 3 | 4 | 3 | 3 | No | No |
| 46 | E1 | Q10 | DeepSeek-V3 | 1 | DA1 | 5 | 5 | 5 | 5 | 5 | No | No |
| 47 | E2 | Q10 | DeepSeek-V3 | 1 | DA1 | 5 | 4 | 4 | 4 | 4 | No | No |
| 48 | E3 | Q10 | DeepSeek-V3 | 1 | DA1 | 4 | 4 | 4 | 4 | 5 | No | No |
| 49 | E4 | Q10 | DeepSeek-V3 | 1 | DA1 | 4 | 5 | 4 | 5 | 5 | No | No |
| 50 | E5 | Q10 | DeepSeek-V3 | 1 | DA1 | 5 | 5 | 5 | 5 | 5 | No | No |
| 51 | E1 | Q10 | DeepSeek-V3 | 2 | DA2 | 3 | 3 | 3 | 3 | 4 | No | No |
| 52 | E2 | Q10 | DeepSeek-V3 | 2 | DA2 | 4 | 4 | 4 | 4 | 5 | No | No |
| 53 | E3 | Q10 | DeepSeek-V3 | 2 | DA2 | 5 | 4 | 4 | 4 | 5 | No | No |
| 54 | E4 | Q10 | DeepSeek-V3 | 2 | DA2 | 4 | 4 | 4 | 4 | 4 | No | No |
| 55 | E5 | Q10 | DeepSeek-V3 | 2 | DA2 | 5 | 4 | 5 | 4 | 3 | No | No |
| 56 | E1 | Q10 | DeepSeek-V3 | 3 | DA3 | 4 | 4 | 4 | 4 | 4 | No | No |
| 57 | E2 | Q10 | DeepSeek-V3 | 3 | DA3 | 4 | 4 | 3 | 4 | 4 | No | No |
| 58 | E3 | Q10 | DeepSeek-V3 | 3 | DA3 | 4 | 5 | 4 | 4 | 5 | No | No |
| 59 | E4 | Q10 | DeepSeek-V3 | 3 | DA3 | 4 | 4 | 4 | 4 | 4 | No | No |
| 60 | E5 | Q10 | DeepSeek-V3 | 3 | DA3 | 4 | 5 | 4 | 3 | 5 | No | No |
| 61 | E1 | Q11 | ChatGPT-5.5 Instant | 1 | CA1 | 5 | 5 | 5 | 4 | 5 | No | No |
| 62 | E2 | Q11 | ChatGPT-5.5 Instant | 1 | CA1 | 4 | 4 | 4 | 4 | 4 | No | No |
| 63 | E3 | Q11 | ChatGPT-5.5 Instant | 1 | CA1 | 5 | 4 | 4 | 4 | 5 | No | No |
| 64 | E4 | Q11 | ChatGPT-5.5 Instant | 1 | CA1 | 4 | 5 | 4 | 5 | 5 | No | No |
| 65 | E5 | Q11 | ChatGPT-5.5 Instant | 1 | CA1 | 5 | 4 | 5 | 5 | 5 | No | No |
| 66 | E1 | Q11 | ChatGPT-5.5 Instant | 2 | CA2 | 4 | 4 | 4 | 4 | 5 | No | No |
| 67 | E2 | Q11 | ChatGPT-5.5 Instant | 2 | CA2 | 5 | 4 | 4 | 5 | 5 | No | No |
| 68 | E3 | Q11 | ChatGPT-5.5 Instant | 2 | CA2 | 5 | 4 | 4 | 4 | 5 | No | No |
| 69 | E4 | Q11 | ChatGPT-5.5 Instant | 2 | CA2 | 4 | 4 | 4 | 4 | 5 | No | No |
| 70 | E5 | Q11 | ChatGPT-5.5 Instant | 2 | CA2 | 5 | 5 | 5 | 5 | 5 | No | No |
| 71 | E1 | Q11 | ChatGPT-5.5 Instant | 3 | CA3 | 4 | 4 | 4 | 4 | 4 | No | No |
| 72 | E2 | Q11 | ChatGPT-5.5 Instant | 3 | CA3 | 5 | 5 | 4 | 5 | 5 | No | No |
| 73 | E3 | Q11 | ChatGPT-5.5 Instant | 3 | CA3 | 5 | 5 | 4 | 4 | 5 | No | No |
| 74 | E4 | Q11 | ChatGPT-5.5 Instant | 3 | CA3 | 4 | 4 | 4 | 5 | 5 | No | No |
| 75 | E5 | Q11 | ChatGPT-5.5 Instant | 3 | CA3 | 5 | 5 | 5 | 5 | 5 | No | No |
| 76 | E1 | Q11 | DeepSeek-V3 | 1 | DA1 | 4 | 4 | 4 | 4 | 4 | No | Yes |
| 77 | E2 | Q11 | DeepSeek-V3 | 1 | DA1 | 5 | 4 | 4 | 4 | 5 | No | Yes |
| 78 | E3 | Q11 | DeepSeek-V3 | 1 | DA1 | 4 | 4 | 4 | 4 | 5 | No | Yes |
| 79 | E4 | Q11 | DeepSeek-V3 | 1 | DA1 | 4 | 5 | 4 | 4 | 5 | No | No |
| 80 | E5 | Q11 | DeepSeek-V3 | 1 | DA1 | 5 | 4 | 5 | 4 | 5 | No | No |
| 81 | E1 | Q11 | DeepSeek-V3 | 2 | DA2 | 2 | 2 | 2 | 3 | 3 | Yes | Yes |
| 82 | E2 | Q11 | DeepSeek-V3 | 2 | DA2 | 4 | 4 | 3 | 4 | 5 | No | Yes |
| 83 | E3 | Q11 | DeepSeek-V3 | 2 | DA2 | 4 | 3 | 3 | 4 | 5 | No | Yes |
| 84 | E4 | Q11 | DeepSeek-V3 | 2 | DA2 | 4 | 3 | 4 | 3 | 3 | No | Yes |
| 85 | E5 | Q11 | DeepSeek-V3 | 2 | DA2 | 4 | 3 | 4 | 3 | 4 | No | Yes |
| 86 | E1 | Q11 | DeepSeek-V3 | 3 | DA3 | 5 | 5 | 5 | 5 | 5 | No | No |
| 87 | E2 | Q11 | DeepSeek-V3 | 3 | DA3 | 5 | 4 | 3 | 4 | 5 | No | Yes |
| 88 | E3 | Q11 | DeepSeek-V3 | 3 | DA3 | 5 | 4 | 4 | 4 | 5 | No | Yes |
| 89 | E4 | Q11 | DeepSeek-V3 | 3 | DA3 | 4 | 5 | 4 | 4 | 5 | No | No |
| 90 | E5 | Q11 | DeepSeek-V3 | 3 | DA3 | 5 | 4 | 5 | 5 | 4 | No | No |
| 91 | E1 | Q12 | ChatGPT-5.5 Instant | 1 | CA1 | 4 | 4 | 4 | 4 | 4 | No | No |
| 92 | E2 | Q12 | ChatGPT-5.5 Instant | 1 | CA1 | 5 | 4 | 3 | 4 | 5 | No | No |
| 93 | E3 | Q12 | ChatGPT-5.5 Instant | 1 | CA1 | 5 | 5 | 4 | 4 | 5 | No | No |
| 94 | E4 | Q12 | ChatGPT-5.5 Instant | 1 | CA1 | 4 | 4 | 4 | 4 | 5 | No | No |
| 95 | E5 | Q12 | ChatGPT-5.5 Instant | 1 | CA1 | 5 | 5 | 5 | 5 | 4 | No | No |
| 96 | E1 | Q12 | ChatGPT-5.5 Instant | 2 | CA2 | 4 | 4 | 4 | 4 | 5 | No | No |
| 97 | E2 | Q12 | ChatGPT-5.5 Instant | 2 | CA2 | 4 | 4 | 3 | 3 | 4 | No | No |
| 98 | E3 | Q12 | ChatGPT-5.5 Instant | 2 | CA2 | 5 | 4 | 4 | 4 | 5 | No | No |
| 99 | E4 | Q12 | ChatGPT-5.5 Instant | 2 | CA2 | 4 | 4 | 4 | 4 | 5 | No | No |
| 100 | E5 | Q12 | ChatGPT-5.5 Instant | 2 | CA2 | 5 | 5 | 5 | 4 | 5 | No | No |
| 101 | E1 | Q12 | ChatGPT-5.5 Instant | 3 | CA3 | 4 | 4 | 4 | 4 | 5 | No | No |
| 102 | E2 | Q12 | ChatGPT-5.5 Instant | 3 | CA3 | 5 | 4 | 3 | 3 | 5 | No | No |
| 103 | E3 | Q12 | ChatGPT-5.5 Instant | 3 | CA3 | 5 | 4 | 3 | 3 | 5 | No | No |
| 104 | E4 | Q12 | ChatGPT-5.5 Instant | 3 | CA3 | 4 | 4 | 4 | 4 | 5 | No | No |
| 105 | E5 | Q12 | ChatGPT-5.5 Instant | 3 | CA3 | 4 | 5 | 5 | 5 | 4 | No | No |
| 106 | E1 | Q12 | DeepSeek-V3 | 1 | DA1 | 5 | 5 | 5 | 5 | 5 | No | No |
| 107 | E2 | Q12 | DeepSeek-V3 | 1 | DA1 | 4 | 4 | 3 | 4 | 5 | No | No |
| 108 | E3 | Q12 | DeepSeek-V3 | 1 | DA1 | 4 | 4 | 4 | 4 | 5 | No | No |
| 109 | E4 | Q12 | DeepSeek-V3 | 1 | DA1 | 4 | 4 | 4 | 5 | 5 | No | No |
| 110 | E5 | Q12 | DeepSeek-V3 | 1 | DA1 | 5 | 5 | 5 | 5 | 5 | No | No |
| 111 | E1 | Q12 | DeepSeek-V3 | 2 | DA2 | 4 | 3 | 3 | 3 | 4 | No | No |
| 112 | E2 | Q12 | DeepSeek-V3 | 2 | DA2 | 4 | 5 | 3 | 3 | 4 | No | No |
| 113 | E3 | Q12 | DeepSeek-V3 | 2 | DA2 | 4 | 4 | 4 | 4 | 4 | No | No |
| 114 | E4 | Q12 | DeepSeek-V3 | 2 | DA2 | 4 | 4 | 4 | 4 | 5 | No | No |
| 115 | E5 | Q12 | DeepSeek-V3 | 2 | DA2 | 5 | 5 | 5 | 4 | 5 | No | No |
| 116 | E1 | Q12 | DeepSeek-V3 | 3 | DA3 | 5 | 5 | 5 | 5 | 5 | No | No |
| 117 | E2 | Q12 | DeepSeek-V3 | 3 | DA3 | 5 | 4 | 3 | 4 | 5 | No | No |
| 118 | E3 | Q12 | DeepSeek-V3 | 3 | DA3 | 5 | 4 | 4 | 4 | 5 | No | No |
| 119 | E4 | Q12 | DeepSeek-V3 | 3 | DA3 | 4 | 5 | 4 | 4 | 5 | No | No |
| 120 | E5 | Q12 | DeepSeek-V3 | 3 | DA3 | 4 | 5 | 4 | 5 | 5 | No | No |
| 121 | E1 | Q13 | ChatGPT-5.5 Instant | 1 | CA1 | 4 | 3 | 3 | 3 | 4 | No | No |
| 122 | E2 | Q13 | ChatGPT-5.5 Instant | 1 | CA1 | 5 | 5 | 4 | 4 | 5 | No | No |
| 123 | E3 | Q13 | ChatGPT-5.5 Instant | 1 | CA1 | 4 | 4 | 4 | 4 | 5 | No | No |
| 124 | E4 | Q13 | ChatGPT-5.5 Instant | 1 | CA1 | 4 | 4 | 4 | 4 | 5 | No | No |
| 125 | E5 | Q13 | ChatGPT-5.5 Instant | 1 | CA1 | 3 | 4 | 5 | 4 | 3 | No | No |
| 126 | E1 | Q13 | ChatGPT-5.5 Instant | 2 | CA2 | 5 | 5 | 5 | 5 | 5 | No | No |
| 127 | E2 | Q13 | ChatGPT-5.5 Instant | 2 | CA2 | 5 | 4 | 4 | 4 | 5 | No | No |
| 128 | E3 | Q13 | ChatGPT-5.5 Instant | 2 | CA2 | 5 | 5 | 4 | 4 | 5 | No | No |
| 129 | E4 | Q13 | ChatGPT-5.5 Instant | 2 | CA2 | 4 | 5 | 4 | 5 | 5 | No | No |
| 130 | E5 | Q13 | ChatGPT-5.5 Instant | 2 | CA2 | 5 | 5 | 5 | 5 | 3 | No | No |
| 131 | E1 | Q13 | ChatGPT-5.5 Instant | 3 | CA3 | 4 | 4 | 4 | 4 | 4 | No | No |
| 132 | E2 | Q13 | ChatGPT-5.5 Instant | 3 | CA3 | 5 | 4 | 3 | 3 | 4 | No | No |
| 133 | E3 | Q13 | ChatGPT-5.5 Instant | 3 | CA3 | 4 | 4 | 4 | 4 | 4 | No | No |
| 134 | E4 | Q13 | ChatGPT-5.5 Instant | 3 | CA3 | 4 | 4 | 4 | 4 | 5 | No | No |
| 135 | E5 | Q13 | ChatGPT-5.5 Instant | 3 | CA3 | 5 | 4 | 5 | 4 | 4 | No | No |
| 136 | E1 | Q13 | DeepSeek-V3 | 1 | DA1 | 5 | 5 | 5 | 5 | 5 | No | No |
| 137 | E2 | Q13 | DeepSeek-V3 | 1 | DA1 | 5 | 5 | 4 | 5 | 5 | No | No |
| 138 | E3 | Q13 | DeepSeek-V3 | 1 | DA1 | 4 | 5 | 4 | 4 | 5 | No | No |
| 139 | E4 | Q13 | DeepSeek-V3 | 1 | DA1 | 5 | 4 | 4 | 4 | 5 | No | No |
| 140 | E5 | Q13 | DeepSeek-V3 | 1 | DA1 | 4 | 4 | 5 | 5 | 4 | No | No |
| 141 | E1 | Q13 | DeepSeek-V3 | 2 | DA2 | 3 | 3 | 3 | 3 | 3 | No | No |
| 142 | E2 | Q13 | DeepSeek-V3 | 2 | DA2 | 4 | 4 | 3 | 4 | 4 | No | No |
| 143 | E3 | Q13 | DeepSeek-V3 | 2 | DA2 | 5 | 4 | 4 | 4 | 5 | No | No |
| 144 | E4 | Q13 | DeepSeek-V3 | 2 | DA2 | 4 | 4 | 4 | 4 | 4 | No | No |
| 145 | E5 | Q13 | DeepSeek-V3 | 2 | DA2 | 3 | 3 | 4 | 4 | 4 | No | No |
| 146 | E1 | Q13 | DeepSeek-V3 | 3 | DA3 | 5 | 5 | 5 | 5 | 5 | No | No |
| 147 | E2 | Q13 | DeepSeek-V3 | 3 | DA3 | 5 | 4 | 4 | 4 | 5 | No | No |
| 148 | E3 | Q13 | DeepSeek-V3 | 3 | DA3 | 5 | 4 | 4 | 4 | 5 | No | No |
| 149 | E4 | Q13 | DeepSeek-V3 | 3 | DA3 | 4 | 5 | 4 | 4 | 5 | No | No |
| 150 | E5 | Q13 | DeepSeek-V3 | 3 | DA3 | 4 | 5 | 5 | 5 | 5 | No | No |
| 151 | E1 | Q14 | ChatGPT-5.5 Instant | 1 | CA1 | 3 | 4 | 3 | 3 | 3 | No | No |
| 152 | E2 | Q14 | ChatGPT-5.5 Instant | 1 | CA1 | 4 | 4 | 3 | 4 | 5 | No | No |
| 153 | E3 | Q14 | ChatGPT-5.5 Instant | 1 | CA1 | 4 | 4 | 4 | 4 | 5 | No | No |
| 154 | E4 | Q14 | ChatGPT-5.5 Instant | 1 | CA1 | 4 | 4 | 4 | 4 | 5 | No | No |
| 155 | E5 | Q14 | ChatGPT-5.5 Instant | 1 | CA1 | 4 | 4 | 5 | 4 | 5 | No | No |
| 156 | E1 | Q14 | ChatGPT-5.5 Instant | 2 | CA2 | 4 | 4 | 3 | 3 | 4 | No | No |
| 157 | E2 | Q14 | ChatGPT-5.5 Instant | 2 | CA2 | 5 | 4 | 3 | 4 | 5 | No | No |
| 158 | E3 | Q14 | ChatGPT-5.5 Instant | 2 | CA2 | 5 | 5 | 4 | 5 | 5 | No | No |
| 159 | E4 | Q14 | ChatGPT-5.5 Instant | 2 | CA2 | 4 | 5 | 4 | 5 | 5 | No | No |
| 160 | E5 | Q14 | ChatGPT-5.5 Instant | 2 | CA2 | 4 | 4 | 5 | 4 | 5 | No | No |
| 161 | E1 | Q14 | ChatGPT-5.5 Instant | 3 | CA3 | 5 | 5 | 5 | 5 | 5 | No | No |
| 162 | E2 | Q14 | ChatGPT-5.5 Instant | 3 | CA3 | 5 | 4 | 4 | 4 | 4 | No | No |
| 163 | E3 | Q14 | ChatGPT-5.5 Instant | 3 | CA3 | 5 | 4 | 4 | 4 | 4 | No | No |
| 164 | E4 | Q14 | ChatGPT-5.5 Instant | 3 | CA3 | 4 | 4 | 4 | 4 | 5 | No | No |
| 165 | E5 | Q14 | ChatGPT-5.5 Instant | 3 | CA3 | 4 | 5 | 5 | 4 | 5 | No | No |
| 166 | E1 | Q14 | DeepSeek-V3 | 1 | DA1 | 5 | 5 | 5 | 5 | 5 | No | No |
| 167 | E2 | Q14 | DeepSeek-V3 | 1 | DA1 | 5 | 4 | 4 | 3 | 5 | No | No |
| 168 | E3 | Q14 | DeepSeek-V3 | 1 | DA1 | 4 | 4 | 4 | 3 | 5 | No | No |
| 169 | E4 | Q14 | DeepSeek-V3 | 1 | DA1 | 4 | 4 | 4 | 4 | 5 | No | No |
| 170 | E5 | Q14 | DeepSeek-V3 | 1 | DA1 | 4 | 5 | 5 | 4 | 5 | No | No |
| 171 | E1 | Q14 | DeepSeek-V3 | 2 | DA2 | 3 | 3 | 3 | 3 | 4 | No | No |
| 172 | E2 | Q14 | DeepSeek-V3 | 2 | DA2 | 4 | 4 | 3 | 4 | 4 | No | No |
| 173 | E3 | Q14 | DeepSeek-V3 | 2 | DA2 | 3 | 3 | 3 | 3 | 4 | No | No |
| 174 | E4 | Q14 | DeepSeek-V3 | 2 | DA2 | 4 | 3 | 4 | 3 | 3 | No | No |
| 175 | E5 | Q14 | DeepSeek-V3 | 2 | DA2 | 3 | 5 | 5 | 3 | 4 | No | No |
| 176 | E1 | Q14 | DeepSeek-V3 | 3 | DA3 | 5 | 5 | 5 | 5 | 5 | No | No |
| 177 | E2 | Q14 | DeepSeek-V3 | 3 | DA3 | 5 | 5 | 4 | 5 | 5 | No | No |
| 178 | E3 | Q14 | DeepSeek-V3 | 3 | DA3 | 5 | 5 | 5 | 4 | 5 | No | No |
| 179 | E4 | Q14 | DeepSeek-V3 | 3 | DA3 | 4 | 5 | 4 | 5 | 5 | No | No |
| 180 | E5 | Q14 | DeepSeek-V3 | 3 | DA3 | 4 | 4 | 5 | 5 | 4 | No | No |
| 181 | E1 | Q15 | ChatGPT-5.5 Instant | 1 | CA1 | 5 | 5 | 5 | 5 | 5 | No | No |
| 182 | E2 | Q15 | ChatGPT-5.5 Instant | 1 | CA1 | 5 | 5 | 4 | 5 | 5 | No | No |
| 183 | E3 | Q15 | ChatGPT-5.5 Instant | 1 | CA1 | 5 | 5 | 5 | 4 | 5 | No | No |
| 184 | E4 | Q15 | ChatGPT-5.5 Instant | 1 | CA1 | 4 | 4 | 4 | 5 | 5 | No | No |
| 185 | E5 | Q15 | ChatGPT-5.5 Instant | 1 | CA1 | 5 | 4 | 5 | 4 | 4 | No | No |
| 186 | E1 | Q15 | ChatGPT-5.5 Instant | 2 | CA2 | 5 | 5 | 5 | 5 | 5 | No | No |
| 187 | E2 | Q15 | ChatGPT-5.5 Instant | 2 | CA2 | 4 | 4 | 3 | 4 | 5 | No | No |
| 188 | E3 | Q15 | ChatGPT-5.5 Instant | 2 | CA2 | 5 | 5 | 5 | 4 | 5 | No | No |
| 189 | E4 | Q15 | ChatGPT-5.5 Instant | 2 | CA2 | 4 | 4 | 4 | 5 | 5 | No | No |
| 190 | E5 | Q15 | ChatGPT-5.5 Instant | 2 | CA2 | 5 | 4 | 5 | 4 | 4 | No | No |
| 191 | E1 | Q15 | ChatGPT-5.5 Instant | 3 | CA3 | 5 | 5 | 5 | 5 | 5 | No | No |
| 192 | E2 | Q15 | ChatGPT-5.5 Instant | 3 | CA3 | 5 | 4 | 3 | 4 | 5 | No | No |
| 193 | E3 | Q15 | ChatGPT-5.5 Instant | 3 | CA3 | 4 | 5 | 4 | 4 | 5 | No | No |
| 194 | E4 | Q15 | ChatGPT-5.5 Instant | 3 | CA3 | 4 | 5 | 4 | 5 | 5 | No | No |
| 195 | E5 | Q15 | ChatGPT-5.5 Instant | 3 | CA3 | 5 | 4 | 5 | 4 | 4 | No | No |
| 196 | E1 | Q15 | DeepSeek-V3 | 1 | DA1 | 4 | 5 | 5 | 5 | 5 | No | No |
| 197 | E2 | Q15 | DeepSeek-V3 | 1 | DA1 | 5 | 5 | 4 | 5 | 5 | No | No |
| 198 | E3 | Q15 | DeepSeek-V3 | 1 | DA1 | 4 | 4 | 4 | 4 | 5 | No | No |
| 199 | E4 | Q15 | DeepSeek-V3 | 1 | DA1 | 4 | 5 | 4 | 5 | 5 | No | No |
| 200 | E5 | Q15 | DeepSeek-V3 | 1 | DA1 | 4 | 4 | 4 | 5 | 5 | No | No |
| 201 | E1 | Q15 | DeepSeek-V3 | 2 | DA2 | 4 | 4 | 4 | 4 | 4 | No | No |
| 202 | E2 | Q15 | DeepSeek-V3 | 2 | DA2 | 5 | 4 | 4 | 4 | 5 | No | No |
| 203 | E3 | Q15 | DeepSeek-V3 | 2 | DA2 | 4 | 4 | 4 | 4 | 5 | No | No |
| 204 | E4 | Q15 | DeepSeek-V3 | 2 | DA2 | 4 | 4 | 4 | 4 | 5 | No | No |
| 205 | E5 | Q15 | DeepSeek-V3 | 2 | DA2 | 4 | 3 | 4 | 4 | 5 | No | No |
| 206 | E1 | Q15 | DeepSeek-V3 | 3 | DA3 | 3 | 3 | 3 | 3 | 3 | No | Yes |
| 207 | E2 | Q15 | DeepSeek-V3 | 3 | DA3 | 5 | 4 | 4 | 4 | 5 | No | No |
| 208 | E3 | Q15 | DeepSeek-V3 | 3 | DA3 | 5 | 5 | 4 | 4 | 5 | No | No |
| 209 | E4 | Q15 | DeepSeek-V3 | 3 | DA3 | 4 | 4 | 4 | 5 | 5 | No | No |
| 210 | E5 | Q15 | DeepSeek-V3 | 3 | DA3 | 3 | 4 | 4 | 4 | 4 | No | No |
| 211 | E1 | Q2 | ChatGPT-5.5 Instant | 1 | CA1 | 3 | 3 | 3 | 3 | 3 | Yes | Yes |
| 212 | E2 | Q2 | ChatGPT-5.5 Instant | 1 | CA1 | 4 | 4 | 3 | 4 | 5 | No | Yes |
| 213 | E3 | Q2 | ChatGPT-5.5 Instant | 1 | CA1 | 4 | 4 | 3 | 4 | 5 | No | Yes |
| 214 | E4 | Q2 | ChatGPT-5.5 Instant | 1 | CA1 | 4 | 4 | 4 | 5 | 5 | No | Yes |
| 215 | E5 | Q2 | ChatGPT-5.5 Instant | 1 | CA1 | 4 | 4 | 5 | 5 | 3 | No | No |
| 216 | E1 | Q2 | ChatGPT-5.5 Instant | 2 | CA2 | 3 | 3 | 3 | 3 | 3 | Yes | Yes |
| 217 | E2 | Q2 | ChatGPT-5.5 Instant | 2 | CA2 | 4 | 4 | 3 | 3 | 3 | No | Yes |
| 218 | E3 | Q2 | ChatGPT-5.5 Instant | 2 | CA2 | 4 | 4 | 3 | 3 | 3 | No | Yes |
| 219 | E4 | Q2 | ChatGPT-5.5 Instant | 2 | CA2 | 4 | 4 | 4 | 5 | 5 | No | Yes |
| 220 | E5 | Q2 | ChatGPT-5.5 Instant | 2 | CA2 | 3 | 4 | 4 | 5 | 4 | No | No |
| 221 | E1 | Q2 | ChatGPT-5.5 Instant | 3 | CA3 | 3 | 3 | 3 | 4 | 4 | Yes | Yes |
| 222 | E2 | Q2 | ChatGPT-5.5 Instant | 3 | CA3 | 4 | 4 | 3 | 4 | 4 | No | Yes |
| 223 | E3 | Q2 | ChatGPT-5.5 Instant | 3 | CA3 | 5 | 4 | 4 | 4 | 4 | No | No |
| 224 | E4 | Q2 | ChatGPT-5.5 Instant | 3 | CA3 | 4 | 5 | 4 | 4 | 5 | No | Yes |
| 225 | E5 | Q2 | ChatGPT-5.5 Instant | 3 | CA3 | 4 | 4 | 4 | 4 | 4 | No | No |
| 226 | E1 | Q2 | DeepSeek-V3 | 1 | DA1 | 4 | 4 | 4 | 4 | 4 | No | No |
| 227 | E2 | Q2 | DeepSeek-V3 | 1 | DA1 | 4 | 4 | 4 | 4 | 4 | No | No |
| 228 | E3 | Q2 | DeepSeek-V3 | 1 | DA1 | 4 | 4 | 4 | 4 | 4 | No | No |
| 229 | E4 | Q2 | DeepSeek-V3 | 1 | DA1 | 4 | 4 | 4 | 4 | 5 | No | No |
| 230 | E5 | Q2 | DeepSeek-V3 | 1 | DA1 | 5 | 5 | 5 | 3 | 5 | No | No |
| 231 | E1 | Q2 | DeepSeek-V3 | 2 | DA2 | 5 | 5 | 5 | 5 | 5 | No | No |
| 232 | E2 | Q2 | DeepSeek-V3 | 2 | DA2 | 4 | 4 | 3 | 4 | 4 | No | No |
| 233 | E3 | Q2 | DeepSeek-V3 | 2 | DA2 | 5 | 4 | 4 | 4 | 4 | No | No |
| 234 | E4 | Q2 | DeepSeek-V3 | 2 | DA2 | 3 | 4 | 4 | 4 | 5 | No | No |
| 235 | E5 | Q2 | DeepSeek-V3 | 2 | DA2 | 3 | 5 | 4 | 4 | 4 | No | No |
| 236 | E1 | Q2 | DeepSeek-V3 | 3 | DA3 | 5 | 5 | 5 | 5 | 5 | No | No |
| 237 | E2 | Q2 | DeepSeek-V3 | 3 | DA3 | 4 | 4 | 3 | 3 | 4 | No | No |
| 238 | E3 | Q2 | DeepSeek-V3 | 3 | DA3 | 5 | 4 | 4 | 4 | 4 | No | No |
| 239 | E4 | Q2 | DeepSeek-V3 | 3 | DA3 | 3 | 4 | 4 | 4 | 5 | No | No |
| 240 | E5 | Q2 | DeepSeek-V3 | 3 | DA3 | 5 | 5 | 5 | 4 | 5 | No | No |
| 241 | E1 | Q3 | ChatGPT-5.5 Instant | 1 | CA1 | 5 | 5 | 5 | 5 | 5 | No | No |
| 242 | E2 | Q3 | ChatGPT-5.5 Instant | 1 | CA1 | 5 | 5 | 4 | 5 | 5 | No | No |
| 243 | E3 | Q3 | ChatGPT-5.5 Instant | 1 | CA1 | 5 | 4 | 4 | 4 | 5 | No | No |
| 244 | E4 | Q3 | ChatGPT-5.5 Instant | 1 | CA1 | 5 | 4 | 4 | 5 | 5 | No | No |
| 245 | E5 | Q3 | ChatGPT-5.5 Instant | 1 | CA1 | 4 | 3 | 4 | 4 | 4 | No | No |
| 246 | E1 | Q3 | ChatGPT-5.5 Instant | 2 | CA2 | 4 | 4 | 4 | 5 | 4 | No | No |
| 247 | E2 | Q3 | ChatGPT-5.5 Instant | 2 | CA2 | 4 | 4 | 4 | 4 | 5 | No | No |
| 248 | E3 | Q3 | ChatGPT-5.5 Instant | 2 | CA2 | 4 | 4 | 4 | 4 | 5 | No | No |
| 249 | E4 | Q3 | ChatGPT-5.5 Instant | 2 | CA2 | 4 | 4 | 4 | 4 | 5 | No | No |
| 250 | E5 | Q3 | ChatGPT-5.5 Instant | 2 | CA2 | 3 | 3 | 5 | 3 | 4 | No | No |
| 251 | E1 | Q3 | ChatGPT-5.5 Instant | 3 | CA3 | 5 | 5 | 5 | 4 | 4 | No | No |
| 252 | E2 | Q3 | ChatGPT-5.5 Instant | 3 | CA3 | 4 | 4 | 4 | 4 | 4 | No | No |
| 253 | E3 | Q3 | ChatGPT-5.5 Instant | 3 | CA3 | 4 | 4 | 4 | 4 | 4 | No | No |
| 254 | E4 | Q3 | ChatGPT-5.5 Instant | 3 | CA3 | 4 | 4 | 4 | 4 | 5 | No | No |
| 255 | E5 | Q3 | ChatGPT-5.5 Instant | 3 | CA3 | 3 | 3 | 5 | 3 | 4 | No | No |
| 256 | E1 | Q3 | DeepSeek-V3 | 1 | DA1 | 5 | 5 | 5 | 5 | 5 | No | No |
| 257 | E2 | Q3 | DeepSeek-V3 | 1 | DA1 | 5 | 5 | 5 | 5 | 5 | No | No |
| 258 | E3 | Q3 | DeepSeek-V3 | 1 | DA1 | 5 | 5 | 4 | 4 | 5 | No | Yes |
| 259 | E4 | Q3 | DeepSeek-V3 | 1 | DA1 | 4 | 4 | 4 | 3 | 5 | No | Yes |
| 260 | E5 | Q3 | DeepSeek-V3 | 1 | DA1 | 4 | 4 | 4 | 5 | 5 | No | Yes |
| 261 | E1 | Q3 | DeepSeek-V3 | 2 | DA2 | 4 | 4 | 4 | 4 | 4 | No | No |
| 262 | E2 | Q3 | DeepSeek-V3 | 2 | DA2 | 5 | 5 | 4 | 5 | 5 | No | No |
| 263 | E3 | Q3 | DeepSeek-V3 | 2 | DA2 | 5 | 4 | 4 | 4 | 5 | No | Yes |
| 264 | E4 | Q3 | DeepSeek-V3 | 2 | DA2 | 3 | 3 | 4 | 3 | 4 | No | Yes |
| 265 | E5 | Q3 | DeepSeek-V3 | 2 | DA2 | 3 | 3 | 5 | 4 | 4 | No | Yes |
| 266 | E1 | Q3 | DeepSeek-V3 | 3 | DA3 | 3 | 3 | 3 | 3 | 3 | No | No |
| 267 | E2 | Q3 | DeepSeek-V3 | 3 | DA3 | 3 | 5 | 3 | 3 | 3 | No | No |
| 268 | E3 | Q3 | DeepSeek-V3 | 3 | DA3 | 3 | 5 | 3 | 3 | 3 | No | Yes |
| 269 | E4 | Q3 | DeepSeek-V3 | 3 | DA3 | 3 | 3 | 4 | 3 | 5 | No | Yes |
| 270 | E5 | Q3 | DeepSeek-V3 | 3 | DA3 | 3 | 4 | 5 | 4 | 5 | No | Yes |
| 271 | E1 | Q4 | ChatGPT-5.5 Instant | 1 | CA1 | 4 | 4 | 4 | 3 | 3 | No | No |
| 272 | E2 | Q4 | ChatGPT-5.5 Instant | 1 | CA1 | 4 | 4 | 4 | 4 | 4 | No | No |
| 273 | E3 | Q4 | ChatGPT-5.5 Instant | 1 | CA1 | 4 | 4 | 4 | 4 | 4 | No | No |
| 274 | E4 | Q4 | ChatGPT-5.5 Instant | 1 | CA1 | 5 | 4 | 4 | 4 | 5 | No | No |
| 275 | E5 | Q4 | ChatGPT-5.5 Instant | 1 | CA1 | 4 | 4 | 4 | 4 | 5 | No | No |
| 276 | E1 | Q4 | ChatGPT-5.5 Instant | 2 | CA2 | 4 | 4 | 4 | 4 | 4 | No | No |
| 277 | E2 | Q4 | ChatGPT-5.5 Instant | 2 | CA2 | 4 | 4 | 4 | 4 | 5 | No | No |
| 278 | E3 | Q4 | ChatGPT-5.5 Instant | 2 | CA2 | 5 | 4 | 4 | 4 | 5 | No | No |
| 279 | E4 | Q4 | ChatGPT-5.5 Instant | 2 | CA2 | 4 | 4 | 4 | 4 | 5 | No | No |
| 280 | E5 | Q4 | ChatGPT-5.5 Instant | 2 | CA2 | 4 | 4 | 4 | 5 | 4 | No | No |
| 281 | E1 | Q4 | ChatGPT-5.5 Instant | 3 | CA3 | 5 | 5 | 5 | 5 | 5 | No | No |
| 282 | E2 | Q4 | ChatGPT-5.5 Instant | 3 | CA3 | 5 | 5 | 4 | 5 | 5 | No | No |
| 283 | E3 | Q4 | ChatGPT-5.5 Instant | 3 | CA3 | 5 | 5 | 4 | 5 | 5 | No | No |
| 284 | E4 | Q4 | ChatGPT-5.5 Instant | 3 | CA3 | 4 | 4 | 4 | 5 | 5 | No | No |
| 285 | E5 | Q4 | ChatGPT-5.5 Instant | 3 | CA3 | 4 | 4 | 5 | 5 | 5 | No | No |
| 286 | E1 | Q4 | DeepSeek-V3 | 1 | DA1 | 4 | 4 | 4 | 3 | 5 | No | No |
| 287 | E2 | Q4 | DeepSeek-V3 | 1 | DA1 | 4 | 3 | 3 | 5 | 5 | No | No |
| 288 | E3 | Q4 | DeepSeek-V3 | 1 | DA1 | 4 | 3 | 3 | 5 | 5 | No | No |
| 289 | E4 | Q4 | DeepSeek-V3 | 1 | DA1 | 4 | 4 | 4 | 4 | 4 | No | No |
| 290 | E5 | Q4 | DeepSeek-V3 | 1 | DA1 | 4 | 5 | 4 | 4 | 5 | No | No |
| 291 | E1 | Q4 | DeepSeek-V3 | 2 | DA2 | 3 | 3 | 3 | 3 | 4 | No | Yes |
| 292 | E2 | Q4 | DeepSeek-V3 | 2 | DA2 | 4 | 4 | 4 | 4 | 5 | No | No |
| 293 | E3 | Q4 | DeepSeek-V3 | 2 | DA2 | 4 | 4 | 4 | 4 | 5 | No | No |
| 294 | E4 | Q4 | DeepSeek-V3 | 2 | DA2 | 4 | 4 | 4 | 4 | 4 | No | No |
| 295 | E5 | Q4 | DeepSeek-V3 | 2 | DA2 | 5 | 5 | 4 | 4 | 3 | No | No |
| 296 | E1 | Q4 | DeepSeek-V3 | 3 | DA3 | 4 | 4 | 4 | 4 | 4 | No | No |
| 297 | E2 | Q4 | DeepSeek-V3 | 3 | DA3 | 4 | 4 | 3 | 4 | 5 | No | No |
| 298 | E3 | Q4 | DeepSeek-V3 | 3 | DA3 | 4 | 4 | 4 | 4 | 5 | No | No |
| 299 | E4 | Q4 | DeepSeek-V3 | 3 | DA3 | 4 | 3 | 4 | 4 | 4 | No | No |
| 300 | E5 | Q4 | DeepSeek-V3 | 3 | DA3 | 4 | 4 | 5 | 4 | 4 | No | No |
| 301 | E1 | Q5 | ChatGPT-5.5 Instant | 1 | CA1 | 3 | 4 | 4 | 3 | 3 | No | No |
| 302 | E2 | Q5 | ChatGPT-5.5 Instant | 1 | CA1 | 5 | 5 | 4 | 5 | 5 | No | No |
| 303 | E3 | Q5 | ChatGPT-5.5 Instant | 1 | CA1 | 5 | 5 | 4 | 5 | 5 | No | No |
| 304 | E4 | Q5 | ChatGPT-5.5 Instant | 1 | CA1 | 5 | 4 | 5 | 4 | 3 | No | No |
| 305 | E5 | Q5 | ChatGPT-5.5 Instant | 1 | CA1 | 4 | 4 | 4 | 4 | 4 | No | No |
| 306 | E1 | Q5 | ChatGPT-5.5 Instant | 2 | CA2 | 4 | 4 | 4 | 4 | 4 | No | No |
| 307 | E2 | Q5 | ChatGPT-5.5 Instant | 2 | CA2 | 4 | 5 | 3 | 5 | 5 | No | No |
| 308 | E3 | Q5 | ChatGPT-5.5 Instant | 2 | CA2 | 4 | 5 | 4 | 4 | 5 | No | No |
| 309 | E4 | Q5 | ChatGPT-5.5 Instant | 2 | CA2 | 4 | 4 | 5 | 4 | 4 | No | No |
| 310 | E5 | Q5 | ChatGPT-5.5 Instant | 2 | CA2 | 5 | 5 | 5 | 5 | 4 | No | No |
| 311 | E1 | Q5 | ChatGPT-5.5 Instant | 3 | CA3 | 4 | 4 | 4 | 4 | 4 | No | No |
| 312 | E2 | Q5 | ChatGPT-5.5 Instant | 3 | CA3 | 4 | 4 | 4 | 4 | 5 | No | No |
| 313 | E3 | Q5 | ChatGPT-5.5 Instant | 3 | CA3 | 4 | 4 | 4 | 4 | 5 | No | No |
| 314 | E4 | Q5 | ChatGPT-5.5 Instant | 3 | CA3 | 4 | 4 | 5 | 4 | 4 | No | No |
| 315 | E5 | Q5 | ChatGPT-5.5 Instant | 3 | CA3 | 5 | 5 | 5 | 5 | 4 | No | No |
| 316 | E1 | Q5 | DeepSeek-V3 | 1 | DA1 | 5 | 5 | 4 | 4 | 5 | No | No |
| 317 | E2 | Q5 | DeepSeek-V3 | 1 | DA1 | 5 | 5 | 4 | 3 | 5 | No | No |
| 318 | E3 | Q5 | DeepSeek-V3 | 1 | DA1 | 5 | 5 | 4 | 4 | 5 | No | No |
| 319 | E4 | Q5 | DeepSeek-V3 | 1 | DA1 | 4 | 4 | 4 | 4 | 4 | No | No |
| 320 | E5 | Q5 | DeepSeek-V3 | 1 | DA1 | 4 | 5 | 5 | 5 | 5 | No | No |
| 321 | E1 | Q5 | DeepSeek-V3 | 2 | DA2 | 4 | 4 | 4 | 4 | 4 | No | No |
| 322 | E2 | Q5 | DeepSeek-V3 | 2 | DA2 | 4 | 4 | 4 | 4 | 5 | No | No |
| 323 | E3 | Q5 | DeepSeek-V3 | 2 | DA2 | 4 | 4 | 4 | 3 | 5 | No | No |
| 324 | E4 | Q5 | DeepSeek-V3 | 2 | DA2 | 4 | 3 | 4 | 4 | 5 | No | Yes |
| 325 | E5 | Q5 | DeepSeek-V3 | 2 | DA2 | 3 | 3 | 4 | 3 | 4 | No | Yes |
| 326 | E1 | Q5 | DeepSeek-V3 | 3 | DA3 | 4 | 3 | 3 | 3 | 5 | Yes | Yes |
| 327 | E2 | Q5 | DeepSeek-V3 | 3 | DA3 | 4 | 4 | 4 | 4 | 5 | No | No |
| 328 | E3 | Q5 | DeepSeek-V3 | 3 | DA3 | 5 | 4 | 4 | 4 | 5 | No | No |
| 329 | E4 | Q5 | DeepSeek-V3 | 3 | DA3 | 4 | 4 | 4 | 4 | 5 | No | No |
| 330 | E5 | Q5 | DeepSeek-V3 | 3 | DA3 | 3 | 3 | 5 | 5 | 5 | No | No |
| 331 | E1 | Q6 | ChatGPT-5.5 Instant | 1 | CA1 | 4 | 4 | 4 | 4 | 5 | No | No |
| 332 | E2 | Q6 | ChatGPT-5.5 Instant | 1 | CA1 | 5 | 5 | 4 | 5 | 5 | No | No |
| 333 | E3 | Q6 | ChatGPT-5.5 Instant | 1 | CA1 | 5 | 4 | 4 | 5 | 5 | No | No |
| 334 | E4 | Q6 | ChatGPT-5.5 Instant | 1 | CA1 | 5 | 4 | 4 | 5 | 5 | No | No |
| 335 | E5 | Q6 | ChatGPT-5.5 Instant | 1 | CA1 | 4 | 4 | 5 | 4 | 3 | No | No |
| 336 | E1 | Q6 | ChatGPT-5.5 Instant | 2 | CA2 | 5 | 5 | 5 | 5 | 5 | No | No |
| 337 | E2 | Q6 | ChatGPT-5.5 Instant | 2 | CA2 | 5 | 5 | 3 | 5 | 5 | No | No |
| 338 | E3 | Q6 | ChatGPT-5.5 Instant | 2 | CA2 | 5 | 5 | 4 | 4 | 5 | No | No |
| 339 | E4 | Q6 | ChatGPT-5.5 Instant | 2 | CA2 | 4 | 4 | 4 | 5 | 5 | No | No |
| 340 | E5 | Q6 | ChatGPT-5.5 Instant | 2 | CA2 | 4 | 4 | 5 | 5 | 3 | No | No |
| 341 | E1 | Q6 | ChatGPT-5.5 Instant | 3 | CA3 | 4 | 4 | 4 | 4 | 5 | No | No |
| 342 | E2 | Q6 | ChatGPT-5.5 Instant | 3 | CA3 | 5 | 5 | 3 | 4 | 5 | No | No |
| 343 | E3 | Q6 | ChatGPT-5.5 Instant | 3 | CA3 | 5 | 5 | 5 | 4 | 5 | No | No |
| 344 | E4 | Q6 | ChatGPT-5.5 Instant | 3 | CA3 | 4 | 4 | 4 | 5 | 5 | No | No |
| 345 | E5 | Q6 | ChatGPT-5.5 Instant | 3 | CA3 | 4 | 4 | 5 | 5 | 4 | No | No |
| 346 | E1 | Q6 | DeepSeek-V3 | 1 | DA1 | 4 | 4 | 4 | 4 | 5 | No | No |
| 347 | E2 | Q6 | DeepSeek-V3 | 1 | DA1 | 4 | 3 | 3 | 4 | 5 | No | No |
| 348 | E3 | Q6 | DeepSeek-V3 | 1 | DA1 | 5 | 4 | 3 | 4 | 5 | No | No |
| 349 | E4 | Q6 | DeepSeek-V3 | 1 | DA1 | 4 | 4 | 4 | 4 | 5 | No | No |
| 350 | E5 | Q6 | DeepSeek-V3 | 1 | DA1 | 5 | 3 | 5 | 5 | 5 | No | No |
| 351 | E1 | Q6 | DeepSeek-V3 | 2 | DA2 | 3 | 3 | 4 | 3 | 3 | No | No |
| 352 | E2 | Q6 | DeepSeek-V3 | 2 | DA2 | 5 | 4 | 4 | 4 | 5 | No | No |
| 353 | E3 | Q6 | DeepSeek-V3 | 2 | DA2 | 5 | 4 | 4 | 4 | 5 | No | No |
| 354 | E4 | Q6 | DeepSeek-V3 | 2 | DA2 | 4 | 4 | 4 | 4 | 4 | No | No |
| 355 | E5 | Q6 | DeepSeek-V3 | 2 | DA2 | 4 | 3 | 5 | 4 | 4 | No | No |
| 356 | E1 | Q6 | DeepSeek-V3 | 3 | DA3 | 4 | 4 | 4 | 4 | 5 | No | No |
| 357 | E2 | Q6 | DeepSeek-V3 | 3 | DA3 | 5 | 5 | 4 | 5 | 5 | No | No |
| 358 | E3 | Q6 | DeepSeek-V3 | 3 | DA3 | 4 | 4 | 4 | 4 | 5 | No | No |
| 359 | E4 | Q6 | DeepSeek-V3 | 3 | DA3 | 4 | 4 | 4 | 4 | 5 | No | No |
| 360 | E5 | Q6 | DeepSeek-V3 | 3 | DA3 | 3 | 3 | 5 | 4 | 5 | No | No |
| 361 | E1 | Q7 | ChatGPT-5.5 Instant | 1 | CA1 | 5 | 5 | 5 | 4 | 5 | No | No |
| 362 | E2 | Q7 | ChatGPT-5.5 Instant | 1 | CA1 | 5 | 3 | 3 | 4 | 5 | No | No |
| 363 | E3 | Q7 | ChatGPT-5.5 Instant | 1 | CA1 | 5 | 4 | 4 | 4 | 5 | No | No |
| 364 | E4 | Q7 | ChatGPT-5.5 Instant | 1 | CA1 | 4 | 4 | 4 | 4 | 5 | No | No |
| 365 | E5 | Q7 | ChatGPT-5.5 Instant | 1 | CA1 | 4 | 5 | 5 | 4 | 5 | No | No |
| 366 | E1 | Q7 | ChatGPT-5.5 Instant | 2 | CA2 | 5 | 5 | 5 | 4 | 5 | No | No |
| 367 | E2 | Q7 | ChatGPT-5.5 Instant | 2 | CA2 | 4 | 4 | 3 | 4 | 5 | No | No |
| 368 | E3 | Q7 | ChatGPT-5.5 Instant | 2 | CA2 | 5 | 4 | 4 | 4 | 5 | No | No |
| 369 | E4 | Q7 | ChatGPT-5.5 Instant | 2 | CA2 | 5 | 5 | 4 | 5 | 5 | No | No |
| 370 | E5 | Q7 | ChatGPT-5.5 Instant | 2 | CA2 | 4 | 5 | 4 | 5 | 5 | No | No |
| 371 | E1 | Q7 | ChatGPT-5.5 Instant | 3 | CA3 | 4 | 4 | 4 | 4 | 5 | No | No |
| 372 | E2 | Q7 | ChatGPT-5.5 Instant | 3 | CA3 | 5 | 5 | 5 | 5 | 5 | No | No |
| 373 | E3 | Q7 | ChatGPT-5.5 Instant | 3 | CA3 | 5 | 5 | 4 | 4 | 5 | No | No |
| 374 | E4 | Q7 | ChatGPT-5.5 Instant | 3 | CA3 | 4 | 4 | 4 | 4 | 5 | No | No |
| 375 | E5 | Q7 | ChatGPT-5.5 Instant | 3 | CA3 | 4 | 4 | 4 | 5 | 5 | No | No |
| 376 | E1 | Q7 | DeepSeek-V3 | 1 | DA1 | 4 | 4 | 4 | 4 | 5 | No | No |
| 377 | E2 | Q7 | DeepSeek-V3 | 1 | DA1 | 5 | 4 | 4 | 4 | 5 | No | No |
| 378 | E3 | Q7 | DeepSeek-V3 | 1 | DA1 | 5 | 4 | 4 | 4 | 5 | No | No |
| 379 | E4 | Q7 | DeepSeek-V3 | 1 | DA1 | 4 | 4 | 4 | 4 | 5 | No | No |
| 380 | E5 | Q7 | DeepSeek-V3 | 1 | DA1 | 3 | 3 | 4 | 4 | 5 | No | No |
| 381 | E1 | Q7 | DeepSeek-V3 | 2 | DA2 | 4 | 4 | 4 | 3 | 3 | No | No |
| 382 | E2 | Q7 | DeepSeek-V3 | 2 | DA2 | 3 | 3 | 3 | 3 | 5 | No | No |
| 383 | E3 | Q7 | DeepSeek-V3 | 2 | DA2 | 4 | 4 | 4 | 4 | 5 | No | No |
| 384 | E4 | Q7 | DeepSeek-V3 | 2 | DA2 | 4 | 3 | 4 | 4 | 3 | No | No |
| 385 | E5 | Q7 | DeepSeek-V3 | 2 | DA2 | 4 | 3 | 4 | 3 | 4 | No | No |
| 386 | E1 | Q7 | DeepSeek-V3 | 3 | DA3 | 5 | 5 | 5 | 5 | 5 | No | No |
| 387 | E2 | Q7 | DeepSeek-V3 | 3 | DA3 | 4 | 4 | 4 | 4 | 5 | No | No |
| 388 | E3 | Q7 | DeepSeek-V3 | 3 | DA3 | 5 | 4 | 4 | 4 | 5 | No | No |
| 389 | E4 | Q7 | DeepSeek-V3 | 3 | DA3 | 4 | 4 | 4 | 4 | 5 | No | No |
| 390 | E5 | Q7 | DeepSeek-V3 | 3 | DA3 | 5 | 5 | 5 | 4 | 4 | No | No |
| 391 | E1 | Q8 | ChatGPT-5.5 Instant | 1 | CA1 | 4 | 4 | 4 | 4 | 5 | No | No |
| 392 | E2 | Q8 | ChatGPT-5.5 Instant | 1 | CA1 | 5 | 5 | 4 | 5 | 5 | No | No |
| 393 | E3 | Q8 | ChatGPT-5.5 Instant | 1 | CA1 | 4 | 4 | 4 | 4 | 5 | No | No |
| 394 | E4 | Q8 | ChatGPT-5.5 Instant | 1 | CA1 | 4 | 4 | 4 | 5 | 5 | No | No |
| 395 | E5 | Q8 | ChatGPT-5.5 Instant | 1 | CA1 | 4 | 5 | 5 | 5 | 5 | No | No |
| 396 | E1 | Q8 | ChatGPT-5.5 Instant | 2 | CA2 | 5 | 5 | 5 | 4 | 5 | No | No |
| 397 | E2 | Q8 | ChatGPT-5.5 Instant | 2 | CA2 | 4 | 4 | 3 | 4 | 5 | No | No |
| 398 | E3 | Q8 | ChatGPT-5.5 Instant | 2 | CA2 | 5 | 5 | 4 | 4 | 5 | No | No |
| 399 | E4 | Q8 | ChatGPT-5.5 Instant | 2 | CA2 | 4 | 5 | 4 | 5 | 5 | No | No |
| 400 | E5 | Q8 | ChatGPT-5.5 Instant | 2 | CA2 | 4 | 5 | 5 | 5 | 4 | No | No |
| 401 | E1 | Q8 | ChatGPT-5.5 Instant | 3 | CA3 | 5 | 5 | 5 | 4 | 5 | No | No |
| 402 | E2 | Q8 | ChatGPT-5.5 Instant | 3 | CA3 | 4 | 4 | 4 | 4 | 4 | No | No |
| 403 | E3 | Q8 | ChatGPT-5.5 Instant | 3 | CA3 | 5 | 5 | 5 | 5 | 5 | No | No |
| 404 | E4 | Q8 | ChatGPT-5.5 Instant | 3 | CA3 | 4 | 4 | 4 | 4 | 5 | No | No |
| 405 | E5 | Q8 | ChatGPT-5.5 Instant | 3 | CA3 | 4 | 4 | 4 | 5 | 4 | No | No |
| 406 | E1 | Q8 | DeepSeek-V3 | 1 | DA1 | 3 | 3 | 3 | 3 | 5 | Yes | Yes |
| 407 | E2 | Q8 | DeepSeek-V3 | 1 | DA1 | 4 | 3 | 3 | 3 | 5 | Yes | Yes |
| 408 | E3 | Q8 | DeepSeek-V3 | 1 | DA1 | 3 | 3 | 3 | 3 | 4 | Yes | Yes |
| 409 | E4 | Q8 | DeepSeek-V3 | 1 | DA1 | 4 | 3 | 4 | 4 | 3 | Yes | Yes |
| 410 | E5 | Q8 | DeepSeek-V3 | 1 | DA1 | 3 | 4 | 3 | 4 | 5 | Yes | Yes |
| 411 | E1 | Q8 | DeepSeek-V3 | 2 | DA2 | 3 | 3 | 3 | 3 | 5 | No | Yes |
| 412 | E2 | Q8 | DeepSeek-V3 | 2 | DA2 | 3 | 3 | 3 | 3 | 4 | No | Yes |
| 413 | E3 | Q8 | DeepSeek-V3 | 2 | DA2 | 4 | 4 | 3 | 4 | 4 | No | No |
| 414 | E4 | Q8 | DeepSeek-V3 | 2 | DA2 | 4 | 3 | 4 | 4 | 3 | No | Yes |
| 415 | E5 | Q8 | DeepSeek-V3 | 2 | DA2 | 3 | 3 | 3 | 4 | 4 | No | Yes |
| 416 | E1 | Q8 | DeepSeek-V3 | 3 | DA3 | 5 | 5 | 5 | 4 | 5 | No | No |
| 417 | E2 | Q8 | DeepSeek-V3 | 3 | DA3 | 3 | 3 | 3 | 3 | 5 | No | No |
| 418 | E3 | Q8 | DeepSeek-V3 | 3 | DA3 | 4 | 4 | 3 | 4 | 5 | No | No |
| 419 | E4 | Q8 | DeepSeek-V3 | 3 | DA3 | 4 | 4 | 4 | 4 | 5 | No | No |
| 420 | E5 | Q8 | DeepSeek-V3 | 3 | DA3 | 4 | 4 | 3 | 4 | 4 | No | No |
| 421 | E1 | Q9 | ChatGPT-5.5 Instant | 1 | CA1 | 3 | 4 | 3 | 4 | 4 | No | No |
| 422 | E2 | Q9 | ChatGPT-5.5 Instant | 1 | CA1 | 5 | 5 | 3 | 5 | 5 | No | No |
| 423 | E3 | Q9 | ChatGPT-5.5 Instant | 1 | CA1 | 4 | 4 | 4 | 4 | 5 | No | No |
| 424 | E4 | Q9 | ChatGPT-5.5 Instant | 1 | CA1 | 4 | 4 | 4 | 4 | 5 | No | No |
| 425 | E5 | Q9 | ChatGPT-5.5 Instant | 1 | CA1 | 4 | 5 | 5 | 4 | 5 | No | No |
| 426 | E1 | Q9 | ChatGPT-5.5 Instant | 2 | CA2 | 4 | 4 | 4 | 4 | 4 | No | No |
| 427 | E2 | Q9 | ChatGPT-5.5 Instant | 2 | CA2 | 5 | 5 | 5 | 5 | 5 | No | No |
| 428 | E3 | Q9 | ChatGPT-5.5 Instant | 2 | CA2 | 5 | 4 | 4 | 4 | 5 | No | No |
| 429 | E4 | Q9 | ChatGPT-5.5 Instant | 2 | CA2 | 4 | 4 | 4 | 4 | 5 | No | No |
| 430 | E5 | Q9 | ChatGPT-5.5 Instant | 2 | CA2 | 5 | 5 | 5 | 4 | 5 | No | No |
| 431 | E1 | Q9 | ChatGPT-5.5 Instant | 3 | CA3 | 5 | 5 | 5 | 4 | 5 | No | No |
| 432 | E2 | Q9 | ChatGPT-5.5 Instant | 3 | CA3 | 5 | 5 | 4 | 3 | 5 | No | No |
| 433 | E3 | Q9 | ChatGPT-5.5 Instant | 3 | CA3 | 5 | 4 | 4 | 4 | 5 | No | No |
| 434 | E4 | Q9 | ChatGPT-5.5 Instant | 3 | CA3 | 4 | 5 | 4 | 4 | 5 | No | No |
| 435 | E5 | Q9 | ChatGPT-5.5 Instant | 3 | CA3 | 5 | 5 | 5 | 5 | 5 | No | No |
| 436 | E1 | Q9 | DeepSeek-V3 | 1 | DA1 | 5 | 5 | 5 | 4 | 5 | No | No |
| 437 | E2 | Q9 | DeepSeek-V3 | 1 | DA1 | 4 | 3 | 3 | 4 | 5 | No | No |
| 438 | E3 | Q9 | DeepSeek-V3 | 1 | DA1 | 5 | 4 | 4 | 4 | 5 | No | No |
| 439 | E4 | Q9 | DeepSeek-V3 | 1 | DA1 | 4 | 4 | 4 | 4 | 5 | No | No |
| 440 | E5 | Q9 | DeepSeek-V3 | 1 | DA1 | 5 | 5 | 5 | 5 | 5 | No | No |
| 441 | E1 | Q9 | DeepSeek-V3 | 2 | DA2 | 3 | 3 | 3 | 3 | 4 | No | No |
| 442 | E2 | Q9 | DeepSeek-V3 | 2 | DA2 | 4 | 4 | 4 | 4 | 5 | No | No |
| 443 | E3 | Q9 | DeepSeek-V3 | 2 | DA2 | 4 | 4 | 3 | 4 | 5 | No | No |
| 444 | E4 | Q9 | DeepSeek-V3 | 2 | DA2 | 4 | 3 | 4 | 4 | 5 | No | No |
| 445 | E5 | Q9 | DeepSeek-V3 | 2 | DA2 | 4 | 4 | 5 | 4 | 5 | No | No |
| 446 | E1 | Q9 | DeepSeek-V3 | 3 | DA3 | 4 | 4 | 4 | 4 | 5 | No | No |
| 447 | E2 | Q9 | DeepSeek-V3 | 3 | DA3 | 5 | 5 | 4 | 4 | 5 | No | No |
| 448 | E3 | Q9 | DeepSeek-V3 | 3 | DA3 | 5 | 4 | 3 | 4 | 5 | No | No |
| 449 | E4 | Q9 | DeepSeek-V3 | 3 | DA3 | 4 | 4 | 4 | 5 | 5 | No | No |
| 450 | E5 | Q9 | DeepSeek-V3 | 3 | DA3 | 4 | 4 | 5 | 4 | 4 | No | No |

**Abbreviations**: AI, artificial intelligence; HPV, human papillomavirus.

**Supplementary Table S3. GEE sensitivity analysis of expert ratings**

| **Outcome** | **Adjusted mean difference ChatGPT-5.5 Instant − DeepSeek-V3 (95% CI)** | **Robust SE** | **P value** | **FDR-adjusted P** |
| --- | --- | --- | --- | --- |
| Accuracy | 0.25 (0.11 to 0.39) | 0.07 | <0.001 | 0.002 |
| Safety | 0.27 (0.07 to 0.47) | 0.10 | 0.008 | 0.013 |
| Guideline concordance | 0.16 (-0.01 to 0.33) | 0.09 | 0.071 | 0.089 |
| Completeness | 0.22 (0.07 to 0.37) | 0.08 | 0.004 | 0.010 |
| Understandability | -0.02 (-0.16 to 0.12) | 0.07 | 0.807 | 0.807 |
| Composite score | 0.18 (0.04 to 0.31) | 0.07 | 0.012 | Not applicable |

Note. Generalized estimating equations with a Gaussian family and exchangeable working correlation were fitted to all 450 expert-rating records. Question was used as the clustering unit, thereby retaining the three repeated generations and five expert ratings within each question, and expert identity was included as a fixed effect to account for systematic rater differences. Positive estimates favour ChatGPT-5.5 Instant. Robust standard errors are reported. Benjamini–Hochberg correction was applied across the five individual Likert domains; the composite score was analysed separately.

**Supplementary Table S4. Exploratory GEE sensitivity analysis of expert-level binary safety outcomes**

| **Outcome** | **Odds ratio, ChatGPT-5.5 Instant vs DeepSeek-V3 (95% CI)** | **P value** |
| --- | --- | --- |
| Major error | 0.43 (0.05 to 4.15) | 0.468 |
| Potential harm | 0.30 (0.03 to 3.01) | 0.309 |
| No major error or potential harm | 3.29 (0.33 to 32.69) | 0.309 |

Note. Exploratory logistic GEE models used question as the clustering unit and included expert identity as a fixed effect. The event counts were sparse, resulting in wide confidence intervals; therefore, the AI-response-level majority-vote analysis in Revised Table 4 should be considered the primary binary safety analysis.

**Supplementary Table S5. Standardized prompt and AI model operating settings**

**Panel A. Prompt structure and model operating settings**

| **Item** | **Specification** |
| --- | --- |
| **Prompt language** | Chinese. |
| **Prompting approach** | Standardized zero-shot prompting. Each session contained only the corresponding patient-style question. No example answer, follow-up prompt, or previous conversational context was supplied. |
| **Full prompt format** | [Corresponding patient-style question in Chinese]. The complete set of 15 prompts and their English translations is provided in Panel B. |
| **Models and platform modes** | ChatGPT-5.5 Instant on the ChatGPT platform and DeepSeek-V3 in Fast mode on the DeepSeek-V3 platform. |
| **Data-generation period** | 15 May 2026 to 30 May 2026. |
| **Internet access** | Internet-connected mode was enabled throughout response generation on both platforms. |
| **Independent-session procedure** | Each question was submitted in a newly opened conversation. For every repeated generation, a separate new conversation was opened and the previous conversation was deleted to minimize carry-over from prior dialogue or cross-conversation memory. |
| **Repeated generations** | Each of the 15 questions was submitted three times independently to each model, producing 45 responses per model and 90 AI-generated responses in total. |
| **Sampling and platform parameters** | The default consumer-platform settings were used. No user-configurable temperature, top-p, seed, or other sampling parameters were manually altered or recorded. |
| **Use of previous context** | No intentionally retained dialogue history or model-specific conversational context was used between runs. |
| **Response handling** | The generated responses were retained as study materials and coded after removing explicit model-source identifiers before expert evaluation. |
| **Evaluation language** | Experts evaluated the original Chinese AI-generated responses. English translations were prepared only for reporting in the supplementary material. |
| **Model-update control** | All responses were generated within the predefined 16-day period. Possible platform-side model updates during this interval could not be independently verified. |

**Panel B. Complete patient-style prompts submitted to both models**

| **No.** | **Original Chinese prompt** | **English translation** |
| --- | --- | --- |
| **Q1** | 我检测出高危型HPV阳性。这是否意味着我将来一定会患宫颈癌？ | I tested positive for high-risk HPV. Does this mean I will definitely develop cervical cancer in the future? |
| **Q2** | HPV 16型或18型阳性严重吗？我一定需要做阴道镜检查吗？ | Is HPV type 16 or 18 positivity serious? Do I definitely need colposcopy? |
| **Q3** | 我是高危型HPV阳性，但TCT结果正常。我需要治疗吗？应该什么时候复查？ | I am positive for high-risk HPV, but my TCT result is normal. Do I need treatment, and when should I be retested? |
| **Q4** | 如果HPV持续阳性、一直不转阴，我该怎么办？有没有药物可以彻底清除HPV？ | What should I do if HPV remains positive and does not clear? Is there any medicine that can completely eliminate HPV? |
| **Q5** | 医生说我有ASC-US或LSIL。这与HPV感染有什么关系？严重吗？ | My doctor said I have ASC-US or LSIL. How is this related to HPV infection, and is it serious? |
| **Q6** | HPV感染可以自行清除吗？通常需要多长时间？ | Can HPV infection clear on its own? How long does it usually take? |
| **Q7** | HPV感染后还能发生性行为吗？会传染给伴侣吗？需要使用安全套吗？ | Can I have sex after HPV infection? Will it be transmitted to my partner, and should we use condoms? |
| **Q8** | 我的伴侣需要做HPV检测或接受治疗吗？ | Does my partner need HPV testing or treatment? |
| **Q9** | 如果我已经感染了HPV，接种HPV疫苗还有意义吗？ | Is HPV vaccination still useful if I am already infected with HPV? |
| **Q10** | HPV感染会影响备孕、妊娠或胎儿吗？ | Will HPV infection affect pregnancy planning, pregnancy, or the fetus? |
| **Q11** | 如果在妊娠期间发现HPV阳性，我该怎么办？可以治疗吗？ | What should I do if HPV is detected during pregnancy? Can it be treated? |
| **Q12** | HPV阳性后，日常生活中需要注意什么？熬夜、压力和免疫力会有影响吗？ | What should I pay attention to in daily life after testing positive for HPV? Do staying up late, stress, and immunity matter? |
| **Q13** | CIN1、CIN2和CIN3分别是什么意思？都需要手术吗？ | What do CIN1, CIN2, and CIN3 mean? Do they all require surgery? |
| **Q14** | 如果我有性交后出血、异常阴道出血或异常阴道分泌物，应该怎么办？ | What should I do if I have bleeding after sex, abnormal vaginal bleeding, or abnormal vaginal discharge? |
| **Q15** | 我非常焦虑，担心HPV会发展成癌症。现在首先应该做什么？ | I am very anxious and worried that HPV will become cancer. What should I do first? |

**Note.** The same 15 prompts were submitted independently to both models. Each prompt was entered three times per model in a separate new conversation. TCT, ThinPrep cytologic test; HPV, human papillomavirus; ASC-US, atypical squamous cells of undetermined significance; LSIL, low-grade squamous intraepithelial lesion; CIN, cervical intraepithelial neoplasia.
